# A Delphi consensus study to identify current clinically most valuable orthopaedic anatomy components for teaching medical students

**DOI:** 10.1186/1472-6920-14-230

**Published:** 2014-10-23

**Authors:** Meenakshi Swamy, Santosh Venkatachalam, John McLachlan

**Affiliations:** School of Medicine, Pharmacy and Health, Holliday Building, Durham University, Queen’s Campus, University Boulevard, Stockton on Tees, TS17 6BH UK; Department of Orthopaedics, Northumbria Healthcare, North Tyneside General Hospital, Rake Lane, North Shields, Tyne and Wear, NE29 8NH UK

**Keywords:** Delphi, Orthopaedics, Clinical anatomy

## Abstract

**Background:**

Over recent years, wide ranging changes have occurred in undergraduate medical curricula with reduction of hours allocated for teaching anatomy. Anatomy forms the foundation of clinical practice. However, the challenge of acquiring sufficient anatomical knowledge in undergraduate medical education for safe and competent clinical practice remains. The purpose of this study is to identify clinically most valuable orthopaedic anatomy components that are relevant to current clinical practice in order to reinforce anatomy teaching.

**Methods:**

Modified Delphi technique with three rounds involving twenty currently practicing orthopaedic consultants and senior speciality orthopaedic registrars (StR, year six and above) was conducted. Anatomical components applied in corresponding clinical situations were generated from the opinions of this expert panel in the first round and the clinical importance of each of these components were rated with a four point Likert scale in the subsequent two rounds to generate consensus. Percentage agreement was utilised as outcome measure for components rated as considerably/very important with consensus of more than 94%.

**Results:**

Response rates were 90% for the first round and 100% for the next two rounds. After three Delphi rounds, thirty four anatomy components applied in general/ specific clinical conditions and clinical tests were identified as clinically most valuable following iteration.

**Conclusions:**

The findings of this study provide clinicians opinions regarding the current required essential anatomical knowledge for a graduating medical student to apply during their orthopaedic clinical encounters. The information obtained can be utilised to encourage further development of clinical anatomy curriculum reflecting the evolving nature of health care.

## Background

Anatomy is one of the important cornerstones of medicine. A firm foundation of satisfactory anatomical knowledge is essential to perform clinical examinations, reach a diagnosis, interpret images and perform clinical procedures safely and effectively. Medical curricula within the UK have undergone significant changes in recent years, especially following the publication of the General Medical Council’s document, Tomorrow’s Doctors [1]. It now covers a spectrum of pedagogic styles from problem-based learning to systems- based teaching, delivered through lectures, small group classes and/or clinical skills sessions. The majority of medical schools in the UK have moved to system based curricula, with a radical reduction in the number of hours allocated to basic science teaching including anatomy [2, 3]. This reduction of anatomy teaching time appears to be an international trend [2]. Also each institution may define its own core systems and modules [4]. Thus anatomical curricular content varies widely at medical schools in the UK and may indicate a lack of consensus regarding the level of anatomical knowledge required for a junior doctor in the UK.

Literature suggests that concerns have been raised over the decline in the anatomical knowledge required for clinical practice, and this has been the subject of considerable debate [2–6]. It has been proposed that medical errors due to inadequate anatomical knowledge are frequently made by junior doctors leading to a rise in litigation [7]. Medical students at graduation feel that their anatomy knowledge is inadequate [5]. A survey of clinicians showed that a majority of them feel that students do not possess the required anatomical knowledge necessary for safe medical practice [8].

Medical students rotate through different clinical specialities during their clinical training and as foundation year doctors. A sound knowledge of anatomy remains central to understanding disease processes and the principles of management. Thus, it is not only important for medical students to possess an understanding of anatomy of the entire human body but also to know how to be able to apply such knowledge appropriately when required in the context of clinical practice. Although the GMC recommends that clinical and basic sciences should be taught in an integrated way throughout the curriculum, anatomy is predominantly taught in the first two years of medical training [2, 6]. Therefore, the process of learning and teaching in their early years should equip students with the required anatomical knowledge and prepare them for its application as clinicians in the respective specialities. However, due to limited clinical exposure and given the time constraints in the first two years (Phase 1 Medicine), it is challenging for students to be able to learn to apply anatomy in the clinical context. It is also difficult for most anatomists to identify and teach relevant clinical anatomy as they are not involved with patients on a regular basis.

In an attempt to inform anatomy teaching and learning, we conducted a Delphi study to gather currently practising specialist clinicians’ opinions as to what they consider the most relevant anatomical components that the students must be aware of, and to collect information on the clinical situations in which they are commonly applied.

The Delphi approach is an iterative group facilitation technique which seeks to obtain group consensus on the opinions of ‘experts’ [9]. It involves a series of structured questionnaires completed anonymously by experts where responses are summarized and fed back for subsequent rounds of iteration. Since the 1960s, the Delphi method has been used to develop content both in medicine, and in industry, in relation to aspects of knowledge and skills [10]. It has been used to determine the undergraduate medical curriculum for dermatology [11] and also required anatomical knowledge for postgraduate education in emergency medicine [10, 12].

In this study, currently practicing orthopaedic consultants and senior speciality orthopaedic registrars (StR- year six and above) represented a panel of ‘experts’ by reason of their day- to- day involvement with patients and close interaction with medical students and junior doctors. Having had the experience of being medical students, foundation year trainees and speciality registrar trainees, and knowing the need for anatomical knowledge, they provided reflective valuable collective opinion on the anatomical knowledge which is currently expected at the level of a foundation doctor.

The aim of the study was to explore those aspects of orthopaedic anatomy currently most valuable in clinical terms, to reinforce anatomy teaching and learning for medical students.

## Methods

Currently practicing orthopaedic consultants and senior speciality orthopaedic registrars (StR) (year six and above) were invited to participate in the study. A modified Delphi technique with three rounds was employed. In the first round of the study, participants were asked to list up to five clinically most relevant anatomical components that the medical students must be aware of, and their corresponding clinical situations. Responses obtained from the first questionnaire were collated and a second round questionnaire was developed from the initial responses. In the second round, the respondents were asked to rate the clinical importance of each of the anatomical components, with the use of a four point Likert Scale; and to provide comments to support their rating where applicable. After this round, the results were analysed for frequency of responses. The third round questionnaire had the summarized responses with the respondents’ personal score indicated by a cross. It provided the respondents an opportunity to change their response in the light of group opinion and add comments if any.

Ethical approval was obtained from the School of Medicine, Pharmacy and Health, Durham University (ESC2/2013/11).

## Results

Twenty initial first round questionnaires were sent out to invited participants. Eighteen of the twenty participants responded (90%). Response rates from the eighteen participants were 100% for the remaining two rounds.

In this study, ninety three anatomy components applied in 173 clinical situations were generated by the participants in the first round of the Delphi study. These were divided into three categories- anatomy components applied in specific clinical conditions, general anatomy components and anatomy components applied in clinical tests. Each anatomical component was considered clinically most valuable if the opinion was rated as considerably important/very important and consensus was 94% and above after the completion of three rounds. Anatomy components for which consensus were achieved is given in Tables 1, 2 and 3. Some participants justified their Likert scale rating in the free text comments section, for example those who rated considerably/very important commented as commonly seen/limb threatening.

**Table 1:** **Anatomy components applied in specific clinical conditions**

Upper limb	Clinical conditions
**Shoulder**	
Shoulder joint anatomy	a. Fractures
	b. Dislocations
**Elbow**	
Antecubital fossa structures including brachial artery location/ distribution	a. Volkmann’s ischaemic contracture
	b. Paediatric supracondylar fractures
**Forearm**	
1. Compartments of forearm	Compartment syndrome
2. Forearm anatomy	Fractures
3. Distal radius	Colles fracture
**Wrist**	
1. Scaphoid	Fractures
2. Carpal tunnel anatomy	Carpal tunnel syndrome
**Hand**	
Tendons of hand	Flexor sheath infection
**Nerves of upper limb**	
1. Median nerve	Carpal tunnel syndrome
2. Radial nerve	Wrist drop-recognition & management
**Lower limb**	
**Pelvis**	
Pelvic anatomy	Pelvic fracture and shock
**Hip**	
Hip joint anatomy	a. Osteoarthritis (arthroplasty)
	b. Fracture neck of femur
	c. Intracapsular vs extracapsular fracture - treatment
**Thigh**	
Femoral triangle anatomy	Embolus -- ischaemic leg
**Knee**	
Knee Joint anatomy and relationships	a. Knee arthritis
	b. Septic arthritis
Surface marking	Knee aspiration
**Leg**	
Compartments of leg	Compartment syndrome
Ankle -- mortise / ligaments	Ankle fractures
**Spine**	
Cervical spine anatomy	Fractures
Spinal cord	a. Cauda Equina Syndrome
	b. Cord compression

**Table 2:** **General anatomy components**

**Upper limb**	
Basic awareness of osteology.	Identify the bones correctly - fractures of bones
**Lower limb**	
Basic awareness of lower limb osteology	Identify the bones correctly - fractures of bones.
**Others**	
Lung fields	Chest X-ray evaluation- pneumonia/pneumo/hemo thorax
Muscle compartment with nerve supply	Compartment syndrome
Tumour principles	
ATLS principles

**Table 3:** **Anatomy components applied in clinical tests**

**Upper limb**	
Flexor and extensor muscles.	Muscle power testing.
Blood supply of upper limb	Assessing circulation in the upper limb.
Peripheral nerves	Dermatomes, myotomes
Nerve supply of the upper limb.	Assessing neurological function.
**Lower limb**	
Abductors of hip	Trendelenberg test
Sciatic/tibial/Common peroneal nerve anatomy	Foot drop
Blood supply of lower limb	Assessing circulation in the lower limb.
Nerve supply of the lower limb.	a. Assessing neurological function.
	b. Radiculopathies vs peripheral nerve entrapment common and important diagnostic situation
Flexor and extensor muscles	Muscle power testing.

## Discussion

Evidence and experience suggests that sound knowledge of applied anatomy is fundamental to competent clinical practice [2]. This supports anatomy teaching to be of the same high standards as patient safety and care. Moreover, it is imperative to establish how much anatomy knowledge should be acquired by students at different levels in their careers.

The American Association of Clinical Anatomists proposed a clinical anatomy curriculum for medical students of the 21^st^ century in 1996 [13], and the Education Committee of the Anatomical Society of Great Britain and Ireland similarly proposed an anatomy syllabus 2007 [7]. However, neither of these curricula has been fully enacted. Other studies have determined anatomy syllabus in head and neck for undergraduate medical students [14] and specialities like emergency medicine for postgraduate education [10, 12]. To our knowledge, there have been no studies conducted to determine the required anatomical knowledge for practice in different clinical specialities at undergraduate level. However, it is not straightforward to understand the relationship between knowledge and its application in clinical practice since there is variation in individual experiences [5]. Hence, in this study, we have utilised Delphi approach to gather collective opinion from experienced currently practising orthopaedic clinicians to help improve clinically relevant anatomy teaching. Work is under progress for conducting Delphi consensus studies in other specialties. The clinical information gathered can help students to become familiar with the clinical situations in which they are required to apply their anatomical knowledge. Hence, when they would encounter similar situation in clinical practice, it might become easier to retrieve information. Students may be able to understand the significance of acquiring anatomical knowledge better.

In our view, anatomy teaching and learning should progress towards exit outcomes which include diagnosis and management of patients by application of an understanding of anatomy as a basis of clinical practice. The transition from medical students to clinical doctors can be stressful. By incorporating current clinically relevant anatomy, students can learn and retain essential information to make this transition smooth. This study has the advantage that the initial content of identification of anatomy components was obtained from experienced orthopaedic surgeons in the first round rather than being derived by the investigators from standard anatomy text books as in some studies [10, 12]. We also ensured that the participants were practising consultant orthopaedic surgeons or speciality registrars year six/above, so that they have had the benefit of going through the training system and were in a better position to identify clinically relevant anatomy components. This ensured that there is minimal disparity between the opinions of the expert panel and what the students/foundation doctors are exposed to in clinical practice. Hence the list is not extensive but with the focus on most important/essential orthopaedic clinical anatomy content needed for a foundation year doctor.

The emphasis of this study was to determine appropriate essential anatomy content for educating medical students and help students to integrate with the clinical information that they will experience in the respective specialities. The eventual desired outcome is to bridge the gap between anatomy and clinical practice.

The findings of the study will enable students to have firm foundation of clinically focussed anatomy required for orthopaedic practice, which they can build upon during their future training. It can help anatomy teachers to have explicit outcomes which are clinically relevant in current orthopaedic practice and ensure that their own particular interests are not covered in depth. Clinical information can be useful for assessment practices.

### Limitations

The drawbacks of this study include relatively small number of participants. However, literature review suggests that such qualitative Delphi studies can be performed with participant numbers varying from as little as nine [15] to as high as sixty [9, 11]. We have also taken a very high consensus percentage (94-100%) as cut off to overcome the drawback of low participant numbers. This also ensured that the identified item list was not exhaustive***.***

Although this methodology succeeds in deriving the key elements with regards to anatomical knowledge, it does not guide us about the pedagogic approach required to deliver these components. We must also be careful in implementing the results of this study so that the core anatomical knowledge is still maintained without the overall structure of the subject being distorted.

Another potential drawback could be that the majority of the participants were from the north east region. While there is a possibility of introducing bias like regional variations in practice of orthopaedics, there is no strong evidence in literature that this might be the case. The clinical aspects identified in this study are dynamic and are likely to vary over time with progress/change of practice in orthopaedics and may need to be repeated at regular intervals.

## Conclusion

This study has helped to benchmark anatomical knowledge requirements that are most relevant to current orthopaedic clinical practice, and essential in teaching medical students. It can be used to highlight the clinical relevance from early years and render anatomy teaching and learning useful for future clinical practice. Thus it can enable students to gain a better understanding of how anatomy knowledge is applied in clinical practice. The content can be recommended to inform clinical anatomy curricula in the future.
